# Prevalence of and Barriers to Dual-Contraceptive Methods Use among Married Men and Women Living with HIV in India

**DOI:** 10.1155/2011/376432

**Published:** 2011-10-12

**Authors:** Venkatesan Chakrapani, Trace Kershaw, Murali Shunmugam, Peter A. Newman, Deborah H. Cornman, Robert Dubrow

**Affiliations:** ^1^Indian Network for People Living with HIV/AIDS (INP+), 50 (Old # 42/12), Second Main Road, Kalaimagal Nagar, Ekkaduthangal, Chennai 600032, India; ^2^Centre for Sexuality and Health Research and Policy (C-SHaRP), 38, Ground Floor, Rangarajapuram Main Road, Kodambakkam, Chennai 600024, India; ^3^Yale School of Public Health, Yale School of Medicine, 60 College Street, P.O. Box 208034, New Haven, CT 06520-8034, USA; ^4^Factor-Inwentash Faculty of Social Work, University of Toronto, 246 Bloor Street West, Toronto, ON, Canada M5S 1A1; ^5^Center for Health, Intervention, and Prevention, University of Connecticut, 2006 Hillside Road, Unit 1248, Storrs, CT 06269, USA

## Abstract

*Objective*. To describe the prevalence and correlates of dual-contraceptive methods use (condoms *and* an *effective* pregnancy prevention method) and barriers to their use among married persons living with HIV (PLHIV) in India. *Methods*. We conducted a quantitative survey (93 men, 97 women), 25 in-depth interviews, seven focus groups, and five key informant interviews. *Results*. Prevalence of dual- contraceptive method use increased from 5% before HIV diagnosis to 23% after diagnosis (*P* < 0.001). Condoms were the most common contraceptive method, with prevalence increasing from 13% before diagnosis to 92% after diagnosis (*P* < 0.001). Barriers to using noncondom contraceptives were lack of discussion about noncondom contraceptives by health care providers, lack of acceptability of noncondom contraceptives among PLHIV, and lack of involvement of husbands in family planning counseling. *Conclusion*. There is a need for interventions, including training of health care providers, to increase dual-contraceptive methods use among married PLHIV.

## 1. Introduction

The sexual and reproductive health of persons living with HIV (PLHIV) is fundamental to their well-being and that of their partners and children. In 2008, there were an estimated 2.27 million PLHIV in India [1], most of whom were adults in the sexually active and reproductive age range and about two-fifths of whom were women [1]. 

The major risk for HIV transmission in India is sexual intercourse [1], with most married women living with HIV having acquired it from their husband [2, 3]. Because increased availability of antiretroviral treatment and management of opportunistic infections have greatly improved the prognosis for PLHIV in India, married PLHIV in the reproductive age range face important reproductive health and family planning decisions. Couples who desire to initiate a pregnancy require careful counselling regarding conception, antenatal care, and childbirth to minimize the risks of transmitting HIV to children and uninfected partners; couples who do not intend to initiate a pregnancy require effective contraception along with protection against both sexually transmitted infections (STIs) and infection or reinfection with HIV [4]. 

Simultaneous protection against both unwanted pregnancy and STIs/HIV is referred to as dual protection [5]. Theoretically, dual protection can be accomplished by consistent male condom use alone; however, typical use of male condoms (hereafter referred to as condoms) as a contraceptive method results in a one-year cumulative incidence of unintended pregnancy of about 15% [6]. Other contraceptive methods, including male and female sterilization, intrauterine devices (IUDs), and oral hormonal contraceptives, are much more effective than condoms in preventing pregnancy, but do not protect against STIs/HIV [6, 7]. Thus, the most prudent approach to dual protection is the use of dual-contraceptive methods: condom use in combination with a highly *effective* pregnancy prevention method. Although promotion and adoption of dual-contraceptive methods pose more challenges than promotion of a single method (e.g., condoms), in a setting where both unintended pregnancy and STI/HIV transmission are of great concern, adoption of dual-contraceptive methods is necessary for optimal sexual and reproductive health [7].

In 2006, we conducted a mixed-methods study of sexual and reproductive health of PLHIV in India [8]. Here we describe results from this study about prevalence of use of contraceptive methods by married men and women living with HIV, prior to and after their HIV diagnosis, assess factors related to their dual-contraceptive methods use after their HIV diagnosis, and assess barriers to the use of dual-contraceptive methods.

## 2. Materials and Methods

We used a concurrent triangulation mixed-methods design [9], in which we conducted a quantitative survey, qualitative in-depth interviews, focus group discussions (FGDs), and key informant interviews from August to November 2006. The goal of using mixed methods was to provide a comprehensive understanding of the phenomenon of dual-contraceptive methods use. The quantitative component primarily explored the prevalence and correlates of dual-contraceptive methods use, and the qualitative component focused on the experiences of using one or more contraceptive methods and explored barriers to use of a noncondom contraceptive method. 

We recruited heterosexual men and women from district-level PLHIV networks of the Indian Network for People Living with HIV (INP+) in five Indian states (Tamil Nadu, Andhra Pradesh, Maharashtra, West Bengal, and Uttar Pradesh). Eligible participants were known to be HIV positive for at least one year, 18 years of age or older, sexually active in the past 3 months, and able to understand and give consent to the study. The study protocol was reviewed and approved by the Institutional Review Board at the University of Toronto, Canada, and by a community advisory board constituted by INP+. All participants except key informants were paid 250 Indian rupees.

### 2.1. Quantitative Component

We used systematic sampling to recruit every *k*th eligible heterosexual man or woman living with HIV, respectively, who attended INP+ support group meetings and drop-in centres of the participating district-level PLHIV networks. This was a simple approach to essentially obtain a random sample. The value of “*k*” was site specific. Overall, we recruited 100 men and 100 women. In the present study, we restricted the quantitative analyses to those who reported being currently married (93 men, 97 women). 

Using a structured questionnaire, trained interviewers asked participants about their current contraceptive use and their contraceptive use prior to their HIV diagnosis. Participants were asked, “Before you tested positive for HIV, did you or your partner ever used any contraceptive/family planning method?” Participants who answered “yes” were then asked, “Which contraceptive/family planning method did you or your partner use?” and were read the following options: the pill, IUD, condom, spermicide, calendar method, injectables, tubal ligation, and vasectomy. Participants were also asked, “Are you or your partner currently using any contraceptive/family planning method?” Participants who answered “yes” were then asked, “Which contraceptive/family planning methods are you currently using?” and were read the options listed above.

 Participants who reported using the pill (oral hormonal contraception), an IUD, injectables, tubal ligation, or vasectomy were scored as using an *effective* contraceptive (pregnancy prevention) method (known to have greater than 90% effectiveness against pregnancy) [6]. Participants who reported using an *effective* pregnancy prevention method *and *condoms were scored as using a dual-contraceptive method. 

The survey also assessed demographic, medical, and behavioural covariates, including age, gender, alcohol consumption in the past 3 months, age at first sexual intercourse, whether the participant had a casual sex partner (“your sexual partner whom you have not met before having sex or with whom you have had only casual acquaintance”) in the past 3 months, principal reasons for using contraceptives (to prevent unwanted pregnancy, to prevent the risk of transmission of HIV to their partner, to prevent getting STIs, to prevent having an HIV-infected baby, and/or partner's preference for contraceptives), HIV status of spouse, years since HIV diagnosis, whether the participant received post-test HIV counselling, whether the participant was currently on antiretroviral drugs, and last CD4 cell count.

### 2.2. Qualitative Component

We recruited heterosexual PLHIV to participate in in-depth interviews and FGDs using snowball sampling, with PLHIV associated with the participating district-level networks serving as seeds, and purposive sampling, to ensure inclusion of persons with seroconcordant and serodiscordant spouses. In-depth interviews were conducted with 25 PLHIV (14 men, 11 women) who reported currently being married and living with their spouse. The interview included open-ended questions to explore the contexts of unprotected and safer sex, current and past use of condoms and other contraceptives, reasons for use or nonuse of various contraceptive methods, and experiences in using those methods. A total of 7 FGDs were conducted with married PLHIV. Each FGD was single gender: three with men (*n* = 15) and four with women (*n* = 28). Discussions focused on sexual and reproductive health needs, use of contraceptives, and prevention challenges. 

 Key informant interviews were conducted with three physicians providing services to PLHIV, one HIV counsellor, and one PLHIV community leader. A community debriefing meeting was conducted with peer research staff and key PLHIV community leaders where the findings were shared and feedback was obtained as a form of “member checking” to enhance the validity of the findings [10].

### 2.3. Data Analyses

We conducted parallel mixed data analysis [11] in which the quantitative and qualitative data were first analysed separately and then compared and contrasted. 

We described results using frequencies and proportions and used McNemar tests to compare contraception method use prior to and after HIV diagnosis. We used a multivariate logistic regression model with forward selection (using *P* < 0.05 as the entry criterion) to calculate odds ratios (ORs) and corresponding 95% confidence intervals (CIs) for potential demographic, medical, and behavioural correlates of dual-contraceptive methods. The variables considered were age, gender, alcohol consumption in the past 3 months, age at first sexual intercourse, whether the participant had a casual sex partner in the past 3 months, principal reasons for using contraceptives (to prevent unwanted pregnancy, to prevent the risk of transmission of HIV to their partner, to prevent getting STIs, to prevent having an HIV-infected baby, and/or partner's preference for contraceptives), HIV status of spouse, years since HIV diagnosis, whether the participant received posttest HIV counselling, whether the participant was currently on antiretroviral drugs, and last CD4 cell count. To aid in the interpretation of the odds ratio for the CD4 count, the count was divided by 50 so that the odds ratio represented the change in odds for every increase in 50 cells/mL of the CD4 count. Statistical analyses were performed with SPSS 17.

In-depth interviews and FGDs were audiotaped, transcribed verbatim in native languages, and translated into English. Data were explored using narrative thematic analysis and a constant comparative method from grounded theory [12, 13].

## 3. Results

### 3.1. Sample Characteristics

Sociodemographic characteristics of study participants are presented in Table 1. The mean age of the survey sample (93 men, 97 women) was 34.1 ± 5.2 years for men and 28.3 ± 4.3 years for women. Sixty-two percent of the men and 71% of the women did not complete high school. Seventy percent of the men and 92% of the women reported being in HIV-seroconcordant relationships. Most PLHIV reported having disclosed their HIV status to their spouse (90% of men, 94% of women). Three men, but no women, reported having a regular sexual partner other than their spouse in the past 3 months. Thirteen men and two women reported having one or more casual partners in the past 3 months. 

Twenty-five married PLHIV (14 men, 11 women) participated in in-depth interviews (Table 1). Their mean age was 31.3 ± 4.6 years. Half (52%) did not complete high school. Half (52%) were volunteer peer educators or part-time peer outreach workers for district-level PLHIV networks. 

A total of 43 married PLHIV (15 men, 28 women) participated in 7 FGDs (Table 1). The mean age of the FGD participants was 30.3 ± 5.1 years. More than half (58%) did not complete high school. About one-third (37%) were volunteer peer educators or part-time peer outreach workers for district-level PLHIV networks.

### 3.2. Use of Contraceptives among Married PLHIV: Quantitative Findings

Table 2 describes contraceptive use before and after HIV diagnosis among married PLHIV. Condoms were the most commonly reported contraceptive method used by married PLHIV prior to and after their HIV diagnosis, followed by tubal ligation. Whereas only 28% of PLHIV (30% of men, 27% of women) reported using any contraceptive prior to their HIV diagnosis, 95% of PLHIV reported currently using a contraceptive (96% of men, 95% of women). This increase, which was highly significant (*P* < 0.001), was mainly due to condom use increasing from 13% of married PLHIV (15% of men; 11% of women) before HIV diagnosis to 92% (92% of men, 92% of women) after diagnosis (*P* < 0.001).

Use of *any effective* pregnancy prevention method (the pill, an IUD, injectables, or sterilization) increased from 19% of married PLHIV (18% of men, 21% of women) prior to HIV diagnosis to 25% (18% of men, 31% of women) after HIV diagnosis. However, this increase was not statistically significant (*P* = 0.20). The increase in tubal ligation use was statistically significant (*P* < 0.01), whereas the increase in use of the pill was not statistically significant (*P* = 0.33). The other *effective* pregnancy prevention methods (injectables, IUDs, and vasectomy) were used too infrequently to assess change.

### 3.3. Use of Dual-Contraceptive Methods among Married PLHIV: Quantitative Findings

Five percent of married PLHIV reported use of dual-contraceptive methods prior to their HIV diagnosis (4% of men, 5% of women), which significantly increased to 23% of married PLHIV after their diagnosis (15% of men, 30% of women) (*P* < 0.001). Thus, over three-fourths of the participants did not use dual-contraceptive methods following their HIV diagnosis; instead, 70% only used condoms (77% of men, 63% of women), 3% only used an *effective* pregnancy prevention method (3% of men, 2% of women), and 5% used neither (4% of men, 6% of women). 

When we explored demographic, medical, and behavioral factors associated with dual-contraceptive methods use after HIV diagnosis, the final model after forward selection included five variables (Table 3). Married PLHIV were significantly more likely to report using dual-contraceptive methods if they were female (OR = 2.93, 95% CI = 1.29–6.67), had received posttest HIV counseling (OR = 2.96, 95% CI = 1.22–7.19), used contraception to prevent the risk of transmission of HIV to their partner (OR = 2.30, 95% CI = 1.01–5.20), or used contraception due to partner's preference (OR = 2.76, 95% CI = 1.08–7.06). Furthermore, married PLHIV were significantly less likely to report using dual-contraceptive methods if they had a higher CD4 cell count (OR = 0.73 per increase of 50 cells/mL, 95% CI = 0.62–0.87). To explore possible differences between sexes, we conducted stratified analyses. Results showed that higher CD4 count was associated with less use of dual-contraceptive methods among both men (OR = 0.73 per increase of 50 cells/mL, 95% CI = 0.55–0.96) and women (OR = 0.75 per increase of 50 cells/mL, 95% CI = 0.62–0.91). In addition, among women, use of contraception to prevent the risk of transmission of HIV to their partner was associated with dual-contraceptive methods use (OR = 4.48, 95%CI = 1.48, 13.54).

### 3.4. Barriers to the Use of Effective Contraceptive Methods: Qualitative Findings

We identified three key barriers among married PLHIV to using noncondom contraceptive methods—either alone or along with condoms: lack of discussion by health care providers about contraceptives other than condoms, lack of acceptability of noncondom contraceptives to PLHIV due to misconceptions about and overestimation of their side effects, and lack of involvement of husbands in family planning counseling, placing the burden for contraception on women.

#### 3.4.1. Lack of Discussion by Health Care Providers about Contraceptives other than Condoms

Most of the participants across different states reported that the focus of HIV and family planning counseling for PLHIV was exclusively on condoms, with very little discussion of other contraceptive methods. As a man explained:

First they (HIV counselors) say “Use condoms and have safe sex.” They don't go to the next level. For general people, what will they say? “If you are not safe, a baby will be born. To avoid that, use Copper-T (IUD) or pills or undergo (sterilization) operation”…but for us there is no choice. Isn't it? As soon as we go, they will say (mimicking the counselor) “My Lord! Without putting on the cover (condom), don't even take it out (laughs).”

This explanation was consistent with the data from key informant health care providers. Two key informant physicians explained that doctors and counselors may emphasize the use of condoms to the exclusion of other contraceptives. Another physician key informant thought some doctors may not talk about condoms or any contraceptives because they do not want to convey the notion to PLHIV that they can be sexually active. 

Some PLHIV who had been using noncondom contraceptives actually stopped using them in the belief that condoms would be sufficient. An in-depth interview participant (a woman peer counselor) said: “Because we emphasize using condoms even if they already have Copper-T, many then say, “We don't want Copper-T, we don't want pills. We will just use *Nirodh* (condoms).” So, in some cases, emphasis on condom use may discourage people from using other contraceptives along with condoms. 

Some women reported unintended pregnancies because they did not use condoms consistently and/or did not use a noncondom contraceptive. For instance, a woman said: “Once I had doubt and underwent abortion when my husband was not well. (I thought), now I don't need a child—later we will…No, I do not use oral pills or Copper-T.” Adequate information and tailored counselling on effective contraceptive methods might have prevented this unintended conception. 

Tubal ligation for women after the birth of two or three children is commonly practiced in India [14]. A woman with two children who had previously visited a government hospital for her first delivery before she became HIV positive recounted how the content of counseling changed after her HIV diagnosis to focus mainly on condom use: “Four years back, they (gynecologists) talked about Copper-T, pills, this and that; now they focus only on condoms—then operation (tubal ligation).” Thus, in addition to condoms, some health care providers talked about tubal ligation with HIV-positive couples who were seen as having completed their family.

#### 3.4.2. Lack of Acceptability of Noncondom Contraceptives

**Misconceptions about and Overestimation of Side Effects of Oral Contraceptive Pills (OCPs)**

Several participants, both women and men, had misconceptions about using non-condom contraceptives. For example, a woman said that she would not want to take OCPs because she has a “hot” body: “I will tell (my husband), “Oh no! My body is usually “hot.” I will die. Don't even mention to me about those tablets.”

Some women, although they might never have tried OCPs, feared side effects as a result of hearing the accounts of other women who had used OCPs. As a woman said, “I do not have any experience (in taking OCPs), and I shall share what I know. Those who take Mala-D (a brand of OCP) get dizziness, body pain…I do not want to use.” This finding suggests that reported experiences of women in informal social networks may influence attitudes toward OCPs.

**Concerns That Convalescence from Tubal Ligation Will Result in Loss of Income**

Of the 19 women in the quantitative survey who reported having had tubal ligation, ten reported having this procedure after their HIV diagnosis. Of the 11 men who reported that their wives had tubal ligation, two participants indicated that their wives had the procedure following their HIV diagnosis. In focus groups and in-depth interviews, some HIV-positive women did not want to undergo tubal ligation in spite of not wanting to conceive again. For instance, although advised by her doctor to undergo tubal ligation, a woman refused because she thought the procedure would result in loss of wages to her family: “My friends complained of (chronic) stomach ache after the operation…I have to work 24 hours. Only if I work will there be money. I said “I will not do it.” My husband also agreed with me.” Another woman said, “Once I get operated on, I will become weak…then I need to be at home for a minimum of 6 months. We need to have a good diet. We don't have that sort of a luxury.” It appears that fear of inability to work and the perception of the need for prolonged bed rest prevented this woman from undergoing tubal ligation.

Discussions with the HIV-positive peer research staff at the community debriefing meeting revealed that many women were also concerned about the effects of tubal ligation on HIV disease progression. These peer research staff also believed that some health care providers were hesitant to perform tubal ligation for women living with HIV due to fear of contracting HIV themselves.

**Concerns That Copper-T (Type of IUD) and OCPs Are Cumbersome and Inconvenient**

Negative experiences of friends who used Copper-T led some women to avoid it. For instance, a woman explained: “Two of my neighbors got rid of Copper-T since it did not suit them… One can get pain or fever. There are many problems with it.”

A key informant who had been counseling PLHIV on family planning methods explained how difficult it is to “convince” some PLHIV to use contraceptives other than condoms:

We say, “Even if you are on oral pills, you must use condoms when you have sex.” We say the same for Copper-T: “Whenever you have sex, always use condoms”…but many would not agree to put on Copper-T in addition to using condoms since it has to be periodically changed. They will make a lot of fuss—even with doctors. Hence we do not want to talk about it (Copper-T). Even when you talk about pills (in addition to condoms), they would not agree. They will say we are already on “strength pills” (vitamins) and how many more (pills) can we take?

#### 3.4.3. Lack of Involvement of Husbands in Family Planning Counseling

In India, a pregnant wife often resides at her parents' home, so her husband often does not accompany her to antenatal care visits. This was seen as a reason for lack of involvement of husbands in family planning methods. A man with one child explained: “When my wife goes for (antenatal care) visits, they also talk about family control. And they try to convince her to have an operation (tubal ligation). But since I am not going with her, they do not talk about family control with me.”

Also, many men living with HIV reported that vasectomy was not discussed with them. Furthermore, they held the misconception that vasectomy might lead to loss of virility as well as to body weakness, which might reduce their capacity to work.

## 4. Discussion

This mixed-methods investigation suggests that dual-contraceptive methods are not widely used by married men and women living with HIV in India. About one-fourth of participants in the survey reported using dual-contraceptive methods—condom use in combination with an *effective* pregnancy prevention method (OCPs, IUDs, injectables, or sterilization). The main reason for the low prevalence of dual-contraceptive methods use was the low prevalence of the use of *effective* pregnancy prevention methods (25%). This was in contrast to the prevalence of condom use, which increased from 13% before HIV diagnosis to 92% after HIV diagnosis.

We know of no previous studies that examined the prevalence of dual-contraceptive methods use among PLHIV in India. The prevalence of dual-contraceptive methods use among PLHIV in our study (23%) was quite high compared with women in Rwanda (1%) [15] and Malawi (1%) [16] and with men and women in Uganda (4% [17] and 5% [18]), but was lower than that reported among women in Soweto, South Africa (33%) [19], the United States (47%) [20], and Brazil (28%) [21].

We are aware of only two studies that examined change in dual-contraceptive methods use after diagnosis of HIV infection. In Brazil, dual-contraceptive methods use among women increased from 2% before HIV diagnosis to 28% after diagnosis [21]. In Malawi, use among women increased from 0.4% before diagnosis to 1.3% one week after diagnosis [16]. 

Our quantitative results were consistent with the qualitative findings that health care providers restrict their discussion of contraceptives with PLHIV mainly to condoms and that non-condom contraceptives tend to lack acceptability among PLHIV due to misconceptions about their use and overblown concerns about their side effects. These findings indicate the need to educate married PLHIV about the benefits of dual-contraceptive methods and to train health care providers to conduct this education effectively, including how to counter misconceptions and unfounded concerns about non-condom contraceptives. 

The survey identified posttest HIV counseling as a significant correlate of the use of dual-contraceptive methods, suggesting that discussion of dual-contraceptive methods with married PLHIV during post-test counseling should be emphasized. Using contraceptives to prevent HIV transmission to the spouse was also significantly correlated with dual-contraceptive methods use, suggesting that appeals to personal responsibility in counseling and educational materials might be useful.

The very low prevalence of vasectomy reported in the survey was consistent with the qualitative finding of lack of involvement of many husbands in family planning counseling. Furthermore, the association in the survey between dual-contraceptive methods use and partner's preference for contraception suggests that involvement of both partners in contraceptive counseling and decision making around family planning might help to increase dual-contraceptive methods use. 

All contraception, including condoms, is free in India [22]. Furthermore, the Indian government provides monetary incentives for tubal ligation, vasectomy, and IUD insertion [23]. Thus, cost is likely not a barrier to adoption of dual-contraceptive methods use. 

The main advantage of the dual-contraceptive methods approach is its efficacy; however, the main drawback is the potential difficulty in motivating people to use two contraceptive methods [7]. A review article on dual protection argued for the importance of promoting dual protection in spite of this difficulty [24]. It is therefore essential that efficient and effective interventions be developed and implemented to promote the use of dual-contraceptive methods among married PLHIV in India, to protect their own and their partner's health, and to prevent unintended pregnancies and consequent possible HIV infection of the infant [25].

The results of this study need to be considered in relation to its limitations. First, all of our participants were associated with PLHIV networks, and more than 40% of the participants in the qualitative component were volunteer peer educators or part-time peer outreach workers for district-level PLHIV networks. Thus, our sample may have been more educated about HIV and contraceptives than the general population of married PLHIV. Furthermore, our finding that counselling focused on condom use to the exclusion of other contraceptive methods may have been skewed by the high proportion in our sample of peer educators/outreach workers, who were trained to focus their education of PLHIV on condom use. 

Second, we did not query about consistency or concurrency of use of each of the various contraceptive methods either before or after HIV diagnosis. However, we did query about consistency of condom use in the past 3 months (after HIV diagnosis) and reported previously that the prevalence of consistent condom use with regular partners was 69% among men and 73% among women [8], compared to the prevalence of current condom use reported here (not taking consistency into account) of 92% among men and 92% among women. Thus, the prevalence of *consistent* dual-contraceptive methods use was undoubtedly even lower than the 23% prevalence of dual-contraceptive methods use measured with the imperfect methodology used in the current study.

Third, we relied on the participant's self-report of spouse's use of contraceptives. Men may have underreported non-condom contraceptive use among their wives because they were not in direct control of their use (e.g., OCPs) or may not have known about their use (e.g., OCPs or IUDs).

Finally, it is possible that responses were influenced by social desirability bias due to the sensitive nature of discussions of sexual behavior and contraception in India. However, because condom distribution and promotion of safer sex are commonplace in PLHIV networks, study participants, who attended these networks, were accustomed to such discussions.

Despite these limitations, our study has offered useful information for designing interventions to promote use of dual-contraceptive methods among married men and women living with HIV in India.

## 5. Conclusion

There is a need for interventions to promote use of dual-contraceptive methods among married men and women living with HIV in India who do not want a child or who want to postpone childbirth. These interventions should promote the husband's involvement in family planning counselling (including discussion of vasectomy) and should provide tailored couples counselling around the benefits of dual-contraceptive methods use (prevention of infection and pregnancy) and the recommended dual-contraceptive methods (condoms *and* a highly *effective* pregnancy prevention method). PLHIV should be presented with the entire range of contraceptive options, and common misconceptions about various contraceptives should be clarified. HIV health care providers and educators and obstetricians/gynaecologists need to be trained about the sexual and reproductive health needs and rights of PLHIV and to be competent in offering family planning counselling in a nonjudgmental, unbiased, PLHIV-centered manner. There is a need for national guidelines to integrate HIV care and reproductive health services [19].

## Figures and Tables

**Table 1:** Sociodemographic characteristics of study participants.

	Quantitative survey		Qualitative component	
Characteristic	Men (*N* = 93)	Women (*N* = 97)	In-depth Interviews (*N* = 25)	Focus group discussions (*N* = 43)
*Sex*				
Male	93 (100%)	—	14 (56%)	15 (35%)
Female	—	97 (100%)	11 (44%)	28 (65%)
*Age (years)*				
18–29	21 (23%)	58 (60%)	7 (28%)	16 (37%)
30–39	57 (61%)	38 (39%)	17 (68%)	23 (53%)
40–49	15 (16%)	1 (1%)	1 (4%)	4 (9%)
*Education*				
<primary	17 (18%)	31 (32%)	1 (4%)	4 (9%)
Primary (5th STD)	19 (20%)	19 (20%)	6 (24%)	9 (21%)
Elementary (8th STD)	22 (24%)	19 (20%)	6 (24%)	12 (28%)
High school (10th STD)	21 (23%)	20 (21%)	7 (28%)	11 (26%)
Higher secondary (12th STD)	9 (10%)	6 (6%)	0 (0%)	5 (12%)
College or higher	5 (5%)	2 (2%)	5 (20%)	2 (5%)
*Occupation*				
Daily wage laborer	40 (43%)	25 (26%)	2 (8%)	2 (5%)
Private company staff	10 (11%)	7 (7%)	1 (4%)	3 (7%)
Peer educator/outreach worker for PLHIV network	12 (13%)	10 (10%)	13 (52%)	16 (37%)
Self-employed	18 (19%)	3 (3%)	2 (8%)	3 (7%)
Homemaker	0 (0%)	34 (35%)	3 (12%)	12 (28%)
Unemployed	10 (11%)	15 (15%)	2 (8%)	2 (5%)
Other	3 (3%)	3 (3%)	2 (8%)	5 (12%)

**Table 2:** Use of contraceptives before and after HIV diagnosis as reported by married persons living with HIV in India.

Contraceptive use	Before HIV diagnosis		After HIV diagnosis		
	Men (*n* = 93)	Women (*n* = 97)	Men (*n* = 93)	Women (*n* = 97)	*P* value^a^
Any contraceptive method	28 (30.1%)	26 (26.8%)	89 (95.7%)	92 (94.8%)	<0.001
Condom use	14 (15.1%)	11 (11.3%)	86 (92.5%)	89 (91.8%)	<0.001
*Effective pregnancy prevention methods*					
Oral hormonal contraception (“the pill”)	2 (2.2%)	6 (6.2%)	3 (3.2%)	10 (10.3%)	0.33
Intrauterine device	4 (4.3%)	6 (6.2%)	1 (1.1%)	0 (0%)	—^b^
Injectables	1 (1.1%)	1 (1.0%)	0 (0.0%)	1 (1.0%)	—
Tubal ligation	9 (9.7%)	9 (9.3%)	11 (11.8%)	19 (19.6%)	<0.01
Vasectomy	1 (1.1%)	0 (0.0%)	3 (3.2%)	1 (1.0%)	—
Any effective pregnancy prevention method	17 (18.3%)	20 (20.6%)	17 (18.3%)	30 (30.9%)	0.20
Dual-contraceptive methods	4 (4.3%)	5 (5.2%)	14 (15.1%)	29 (29.9%)	<0.001
*Ineffective pregnancy prevention methods*					
Spermicide	1 (1.1%)	0 (0.0%)	0 (0.0%)	0 (0.0%)	—
Calendar method	3 (3.2%)	1 (1.0%)	1 (1.1%)	1 (1.0%)	—

^a^ *P* value for the change in contraceptive use among men and women combined.

^b^Numbers too small to assess change.

**Table 3:** Correlates of dual-contraceptive methods use among married persons living with HIV in India^a^

Variable	Odds ratio	95% confidence interval
*Sex*		
Male	1.00	—
Female	2.93	1.29–6.67
*Posttest HIV counselling*		
No	1.00	—
Yes	2.96	1.22–7.19
*Used contraception to prevent risk of transmission of HIV to partner*		
No	1.00	—
Yes	2.30	1.01–5.20
*Used contraception due to partner's preference*		
No	1.00	—
Yes	2.76	1.08–7.06
Last CD4 cell count (per increase of 50 cells/mL)	0.73	0.62–0.87

^a^Odds ratios and 95% confidence intervals were calculated using a multivariate logistic regression model with forward selection (using *P* < 0.05 as the variable entry criterion).
